# The Perpetual Diamond: Contrast Reversals Along Thin Edges Create the Appearance of Motion in Objects

**DOI:** 10.1177/2041669518815708

**Published:** 2018-12-19

**Authors:** Oliver J. Flynn, Arthur G. Shapiro

**Affiliations:** Department of Psychology, American University, Washington, DC, USA; National Eye Institute, National Institutes of Health, Bethesda, MD, USA; Department of Psychology, American University, Washington, DC, USA; Department of Computer Science, American University, Washington, DC, USA

**Keywords:** contrast, motion, first-order motion, motion illusion, psychophysics, local motion, acuity

## Abstract

The Perpetual Diamond produces motion continuously and unambiguously in one direction despite never physically changing location. The phenomenon consists of a steady, mid-luminance diamond bordered by four thin edge strips and a surrounding background field. The direction of motion is determined by the relative phases of the luminance modulation between the edge strips and the background. Because the motion is generated entirely by changing contrast signals between the edge strips and background, the stimulus is a valuable tool for tests of spatial contrast, temporal contrast, contrast gain, and color contrast. We demonstrate that observers see motion even when the edge strips subtend only seconds of arc on the retina (which is less than the frequently reported 10 minutes of arc) and that perceived motion is due entirely to changes in the difference in contrast phase modulation, independent from the luminance phase.

It is well known that displays that contain shifts in contrast at edges can create the perception of motion (Anstis & Rogers, 1986; Gregory & Heard, 1983; Hock & Gilroy, 2005; Mather, 2006; Shapiro, Charles, & Shear-Heyman, 2005; Shapiro & Knight, 2008). Gregory and Heard’s (1983) *Phenomenal Phenomena* appear to bounce left and right as contrast between the left and right edge bars and the central rectangle modulate in time. Anstis and Rogers (1986) and Mather and Murdoch (1999) demonstrated stimuli that appeared to move continuously in one direction by switching the contrast polarity (either first-order contrast or second-order contrast) each time they moved in reverse. The contrast reversals somehow mask the reversed motion or create their own reversed motion signal. However, in these stimuli, the object itself that appears to move also changes luminance (or texture) constantly over time. Here, we demonstrate a stimulus, which we call the Perpetual Diamond, that neither moves nor changes in luminance or texture, yet it appears to move continuously in one of the four directions.

The stimulus is demonstrated in Movie 1. A static, mid-luminance diamond is surrounded by very thin edge strips and placed on a background. The luminance (or color) of the edges and background modulate sinusoidally in time at 2 Hz. Motion occurs when the temporal phases of the edge modulations are shifted relative to the modulation of the background. So, for instance, if the two top edges of the diamond modulate in quadrature phase ahead of the background and the two bottom edges modulate in quadrature phase behind the background, the diamond appears to move continuously upward. The diamond can be oriented in any cardinal direction, and the motion is just as strong.

**Figure 1.**
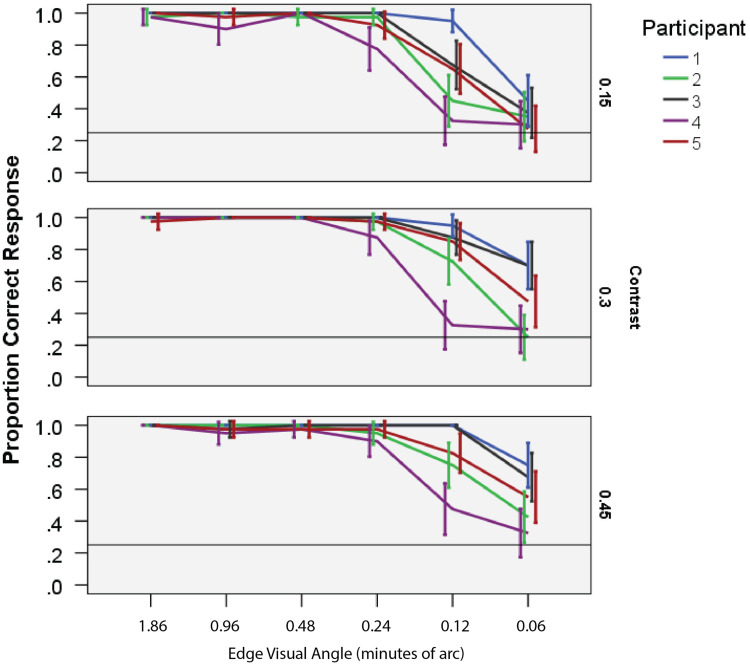
Correct response versus edge-width visual angle for five observers: each panel, a different modulation contrast; each line, a different observer. Observers could reliably detect motion with edge widths as thin as 3.6 seconds of visual angle. The order of individual sensitivity was consistent across contrast levels.

**Movie 1.** **(click to play)**. The basic Perpetual Diamond. The luminance of edges and background modulate in time. Diamond direction is controlled by the phase differences between edge modulations and the background.

**Movie 2.** **(click to play)**. The diamond’s motion is independent of the absolute phase of luminance modulation. The video shows four squares attached to each edge; the luminance phases of the squares can change independently. The diamond’s direction is controlled entirely by manipulating differences in modulation phase between each edge and square.

If we divide the background into four separate rectangles, each bordering one edge strip, we can modulate each out of phase with the others, and the diamond motion is preserved as long as each edge remains in quadrature phase with its respective background. Motion in the Perpetual Diamond is therefore generated along all four edges independently and simultaneously. These four local motion signals are then combined by the visual system to create the perception of a moving object (Scarfe & Johnston, 2011; see Movie 2).

We measured the effect of viewing distance on observers’ ability to detect the diamond motion using a four-alternative forced choice test (all ethical protocols, including informed consent, were followed in accordance with tenets of the Declaration of Helsinki). Observers viewed the stimulus on a calibrated monitor (linearized, mean luminance 50 cd/m^2^) down a long, darkened hallway. We found that observers could detect the motion accurately (above 25% chance level) even when the edges subtended as little as 0.0010° of visual angle (0.06 minutes or 3.6 seconds of arc) (Figure 1). This remarkable acuity (often referred to, perhaps erroneously, as hyperacuity) is consistent with others’ measures (Carney & Klein, 1997; Lee, Wehrhahn, Westheimer, & Kremers, 1995; Wilkinson, Wilson, & Habak, 1998).

The motion may be considered in terms of simple changes in contrast. Indeed, the creation of the Perpetual Diamond stemmed from studies of contrast information not motion. Shapiro and colleagues have been examining and creating perceptual phenomena based on the conflict between absolute and relative stimulus values. The general idea is that the visual system has separate perceptions of luminance and contrast information available in the image (Frazor & Geisler, 2006; Hedjar, Cowardin, & Shapiro, 2018; Shapiro et al., 2004, 2005,; Shapiro & Knight, 2008; Whittle, 2004). Contrast can affect grouping (Shapiro & Hamburger, 2007) and generate motion (Shapiro et al., 2005).

The Perpetual Diamond is a striking motion display that is simple and provides no clues as to its orientation or direction until it is animated. Because the motion is generated through modulating contrast signals alone, the stimuli may be used to test contrast sensitivity in various ways. For example, we suspect that the stimulus may be valuable for future studies of color, by modulating the edges and background along color confusion lines; contrast gain, by varying the modulation amplitude between the edges and background; and motion integration, by increasing the thickness of the edges.

We successfully modeled the direction of motion under a wide variety of conditions, using a slight modification of Challinor and Mather’s (2010) first-order motion model (which is adapted from Adelson & Bergen, 1985), to evenly weight dark and light inputs (Adelson & Bergen, 1985; Challinor & Mather, 2010). Our result suggests that the perceived motion in the Perpetual Diamond, like other reverse phi motion phenomena, corresponds to motion energy available in the stimulus even though the edges and diamond remain in a fixed location.

If an illusion is conceived of as a *perception* that differs from *reality* (whatever those two terms are believed to mean), then the experience of motion in the Perpetual Diamond is not an illusion: As the contrast modulation creates motion energy, the perception of motion corresponds to a property physically present in the stimulus. However, if an illusion is thought to be the result of the brain trying to construct a perceptual reality out of potentially contradictory information (Shapiro & Todorović, 2017), then the Perpetual Diamond is an illusion as one source of stimulus information (the motion energy) conflicts with another source of information (the stationary location of the diamond). The best perceptual story seems to be that the diamond moves yet remains in the same location.

## Authors’ Note

Data can be accessed on request from the corresponding author.
